# Risk Factors of African Swine Fever in Domestic Pigs of the Samara Region, Russian Federation

**DOI:** 10.3389/fvets.2021.723375

**Published:** 2021-08-24

**Authors:** Anastasia A. Glazunova, Fedor I. Korennoy, Timofey A. Sevskikh, Daria A. Lunina, Olga I. Zakharova, Andrei A. Blokhin, Anton K. Karaulov, Andrey E. Gogin

**Affiliations:** ^1^Federal Research Center for Virology and Microbiology, Branch in Samara, Samara, Russia; ^2^Federal Center for Animal Health (FGBI ARRIAH), Vladimir, Russia; ^3^Federal Research Center for Virology and Microbiology, Branch in Nizhny Novgorod, Nizhny Novgorod, Russia; ^4^Federal Research Center for Virology and Microbiology, Pokrov, Russia

**Keywords:** African swine fever, Russian Federation, Samara region, logistic regression, low-biosecurity farms, colocation analysis, animal movement

## Abstract

African swine fever (ASF) is an incurable viral disease of domestic and wild pigs. A large-scale spread of ASF began in Eurasia in 2007 and has affected territories from Belgium to the Far East, occurring as both local- and regional-level epidemics. In 2020, a massive ASF epidemic emerged in the southeastern region of European Russia in the Samara Oblast and included 41 outbreaks of ASF in domestic pigs and 40 cases in wild boar. The Samara Oblast is characterized by a relatively low density of wild boar (0.04–0.05 head/km^2^) and domestic pigs (1.1–1.3 head/km^2^), with a high prevalence of small-scale productions (household farms). This study aims to understand the driving forces of the disease and perform a risk assessment for this region using complex epidemiological analyses. The socioeconomic and environmental factors of the ASF outbreak were explored using Generalized Linear Logistic Regression, where ASF infection status of the Samara Oblast districts was treated as a response variable. Presence of the virus in a district was found to be most significantly (*p* < 0.05) associated with the importation of live pigs from ASF-affected regions of Russia (OR = 371.52; 95% CI: 1.58–87290.57), less significantly (*p* < 0.1) associated with the density of smallholder farms (OR = 2.94; 0.82–10.59), volume of pork products' importation from ASF-affected regions of Russia (OR = 1.01; 1.00–1.02), summary pig population (OR = 1.01; 0.99–1.02), and insignificantly (*p* > 0.1) associated with presence of a common border with an ASF-affected region (OR = 89.2; 0.07–11208.64). No associations were found with the densities of pig and wild boar populations. The colocation analysis revealed no significant concentration of outbreaks in domestic pigs near cases in wild boar or vice versa. These results suggest that outbreaks notified in low biosecurity household farms were mainly associated with the transportation and trade of pigs and pork products from ASF-affected regions of Russia. The findings underline the importance of taking into account animal transportation data while conducting future studies to develop a risk map for the region and the rest of European Russia.

## Introduction

African swine fever (ASF) is one of the most dangerous transboundary diseases of domestic and wild suids and is characterized by high lethality and serious socio-economic consequences due to the lack of a vaccine (1). The largest epidemic of ASF in the history of Eurasia caused by a highly virulent virus of the II p72 genotype began 15 years ago in South Caucasus and since then has spread without the involvement of natural hosts or biological vectors. The pandemic has spread to Middle Eastern countries, North Caucasus, East and West Europe, the Russian Far East, China, and southeastern Asian and Oceanian countries.

**Figure 1:**
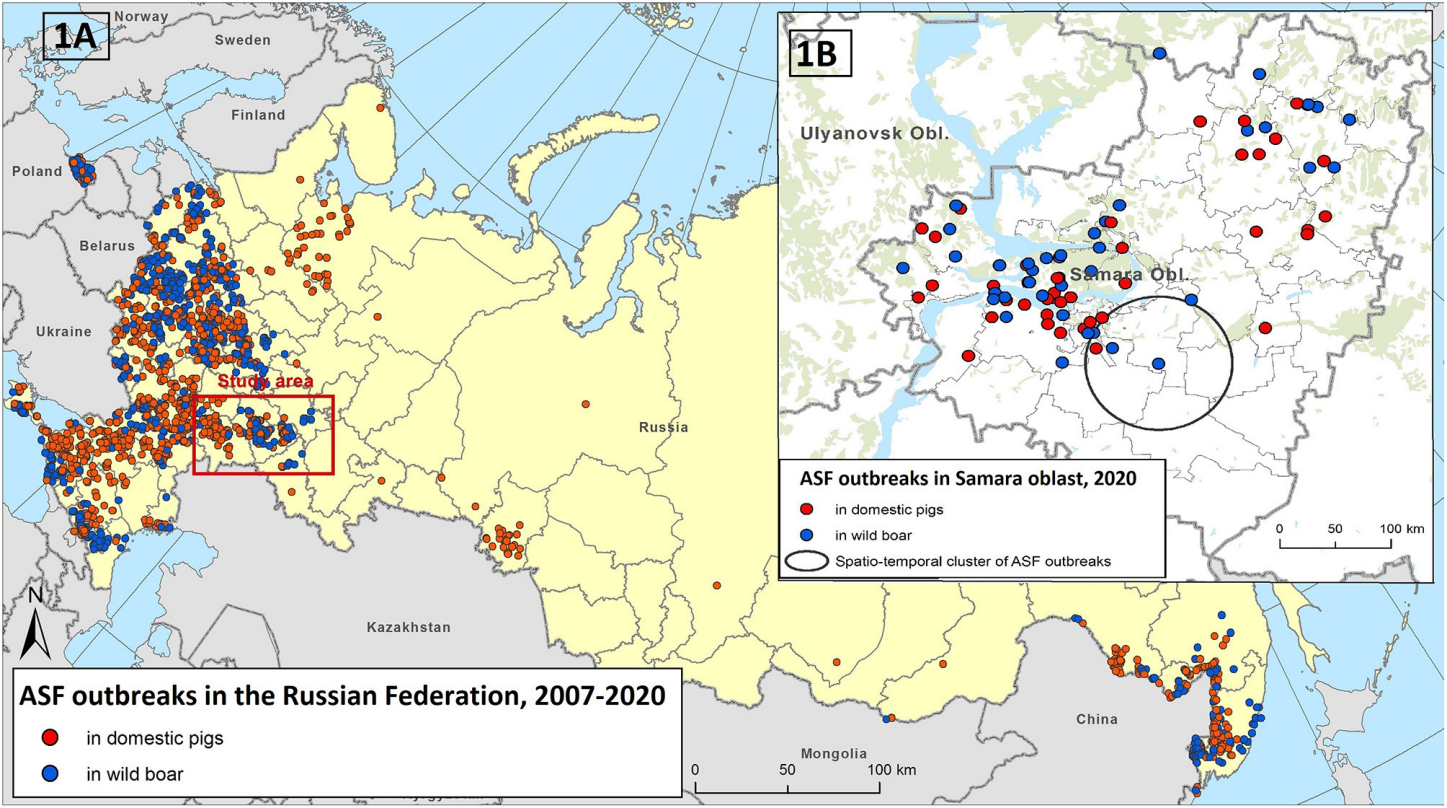
The epidemiological overview of African swine fever (ASF) in the Russian Federation in 2007–2020 **(A)**. The ASF outbreaks in the Samara Oblast in 2020 and revealed spatial-temporal cluster of outbreaks **(B)**.

The spread of ASF in Eurasia has continued since 2007, resulting in local and large-scale epidemics in domestic pig and wild boar populations. ASF has gradually and continuously spread throughout the southern regions of the Russian Federation and has sporadically jumped to regions distant from ASF-affected zones. In 2011, the number of ASF notifications increased significantly in the central regions of the European part of Russia. In 2017, sporadic cases of ASF were reported in the Asian part of Russia, and in 2019, ASF became endemic in the Russian Far East (Figure 1A).

Throughout the Russian Federation, the majority of ASF outbreaks were registered in domestic pigs. As of the end of 2020, 1,074 outbreaks in domestic pigs and 737 cases in wild boar were reported to the World Organization for Animal Health (OIE). A similar pattern was observed in Eastern Europe and Asian countries, especially in countries in which small pig productions are more common than commercial pig farms.

The ASF virus (ASFV) is spread and maintained outside Africa in the absence of the classic sylvatic cycle without (or with limited) participation of pig-associated *Ornithodoros* tick species involving wild boar and domestic pigs as reservoirs of virus (2). For this reason, the epidemiology of ASF outside Africa is complex and varies depending on the availability and type of factors of transmission and the epidemiological reservoirs. Circulation of the virus in Eurasia is maintained via the domestic cycle (when the virus circulates in domestic pigs and pig-derived products) and the wild boar-habitat cycle (when the virus circulates in wildlife without the involvement of ticks). These cycles are not isolated and can overlap, allowing the virus to survive for a long time and spread over long distances (3). The circulation of the virus in the domestic pig sector involves human activity and biosecurity, as it occurs within pig farms. The tendency for ASF to become endemic is common in countries with small-scale pig production units with inadequate biosecurity (4).

The functioning of the cycles of the virus, their interactions, and the establishment of endemicity are dependent on several factors such as the biological properties of both the virus and host and abiotic factors (including environmental and natural conditions and social and economic circumstances). According to previous investigations of ASF epidemics in the Russian Federation, susceptible animals typically become infected after contact with infected animals or contaminated fomites, feed, vehicles, or clothing (5). A matched case-control study conducted in Romania revealed that in addition to proximity to outbreaks in domestic farms, the abundance of wild boar and a short distance between the farm and infected wild boar were significant risk factors for the spread of ASF in commercial and small-scale pig farms (6).

Studying the features of an epidemic and ASF epidemiology in a specific region may improve the understanding of its driving forces, risk factors, and transmission routes, especially when the virus is present in both domestic and wild boar populations. Often, epidemiological investigations do not provide information regarding the exact source and risk factors of introduction of virus and whether the domestic pigs or the wild boar plays the primary role in sustaining and spreading of the ASFV. A better understanding of these factors will help provide an effective solution for the prevention and eradication of ASF.

The Samara Oblast is a region where the virus has rapidly spread within a year affecting large areas, and caused at least 41 outbreaks in domestic pigs and 40 cases in wild boar, which accounted for nearly one-third of all ASF outbreaks in the Russian Federation during 2020 (Figure 1B). Therefore, this region can be used as a model to investigate the role of various epidemic drivers shaping the observed disease spread. In this study, for the first time we use the national surveillance data on animal movements among other potential risk factors, to explain the observed pattern of ASF outbreaks' distribution in domestic pigs and to provide a basis for the further development of an ASF risk model at both national and regional level.

## Materials and Methods

### Data

#### Study Area

The Samara Oblast, a region located in southeast European Russia bordering Kazakhstan in the south, is one of 59 ASF affected regions of the Russian Federation.

The average human population density in the region is ~60 people/km^2^. The human population is concentrated around the administrative center and significantly decreased in the peripheral districts. The Samara Oblast has an advantageous economic-geographical location, as two international transport corridors (North-South and West-East) intersect in this region. The central part of the region and large cities have the highest density of roads. The main agricultural areas are situated in the periphery of the region.

Second-level administrative units termed districts (*n* = 37) were used as spatial units for the assessment of the epidemiological parameters and potential risk factors in this study. Two large cities were excluded as they were statistical outliers for human population density and have no pig husbandry areas. Four districts that have no pig population according to official statistics were also excluded. Therefore, the spatial analysis included 31 districts of the Samara Oblast.

River networks are poorly developed in the southern areas of the Samara Oblast. The main waterbody is the Volga River, which delimits the western part of the region. Forests cover 14% (760,000 ha) of the Samara Oblast, including 25–40% of the central part of the region, 3% of the southern part of the region, and 14% of the northern part of the region. There are 217 wildlife nature reserves and national parks in the region where wild boar hunting is prohibited. Sown areas, reserves, and wildlife parks have lakes and rivers that provide a good habitat for wild boar. At the beginning of 2020, the estimated total number of wild boar in the Samara Oblast was 2,345, and the northern and central parts of the region had the highest densities of wild boar (0.78–1.31 ind. per 1,000 hectares). The areas of the Samara Oblast border the Saratov and Ulyanovsk Oblasts, which were already affected by ASF in 2019.

#### Domestic Pig Sector Characteristics

The agricultural sector of the Samara Oblast is focused mainly on crop production. Most of the meat produced in the region is poultry (60%), while pork accounts for 24% of the meat produced in the region (7). The density of the pig population is relatively low in this region, with most pigs kept in small-scale farms. At the end of 2019, the total number of ASF-susceptible animals was 187,185 pigs, including 84,075 heads in small-scale farms (backyards, for self-consumption), 9,954 in non-specialized commercial farms (from 3 to 3,695 pigs per holding), and 56,342 in large-scale specialized commercial pig farms. In August 2020, the total number of pigs was ~192,000.

A significant proportion of the domestic pigs in this region were contained in holdings with a low level of biosecurity, where restrictive and safety measures were implemented only in emergency situations. At the end of 2020, only 32 pig husbandries had official biosecurity statuses. Overall, 68.75% of households were unprotected, 6.25% had low-level biosecurity, 12.5% had average levels of biosecurity, and 12.5% had high levels of biosecurity.

#### ASF Data

This study used ASF outbreaks data notified by the Russian Federation to OIE (8). According to these data, 41 outbreaks occurred in domestic pigs and 40 cases registered in wild boar in the Samara Oblast in February—December 2020. The exact geographical coordinates, disease start date, and numbers of susceptible and infected animals were reported for each outbreak.

#### Explanatory Factors

Human and pig population data were acquired from the Federal Service of Governmental statistics (9). Data regarding wild boar population were obtained from the Department of Hunting and Fishing (10). Data regarding settlements and smallholder farm distribution were acquired from the official registry of supervised objects and compartments (11). Data regarding the legal movements of pigs and pork products between the Samara Oblast and the regions of Russia that were affected by ASF in 2019 and 2020 were acquired from “Mercury” database—the state information system of Rosselkhoznadzor (12). This system is designed to trace cargo that is monitored by state veterinary services. Data regarding road networks were acquired from the official website of the Samara Oblast Government (13). Data regarding forest areas were extracted from the raster dataset generated from Earth remote sensing system Proba-V from 2000 to 2018 with an original spatial resolution of 100 × 100 m (14).

Table 1 includes the geospatial variables used for the analyses. Supplementary Figures 1–14 (see Supplementary Material) present the distribution maps of these variables in the Samara Oblast. To avoid multicollinearity, all variables in this study were previously tested using the Spearman rank correlation test. The further modeling used only variables with correlation levels |r_s_| ≤ 0.7.

**Table 1:** Geospatial variables.

	**Unit**	**Variable type (as used in regression analysis)**	**Median (minimum–maximum)**	**VIF**
Primary road length	km	Continuous	306.6 (23.3–650.8)	4.06
Road density	km^−1^	Continuous	0.63 (0.32–17.3)	>5
Human population density	Persons/km^2^	Continuous	12.7 (5.7–1428.7)	Excluded as highly correlated
Density of smallholder farms	Holdings/km^2^	Continuous	0.5 (0–5.3)	1.41
Domestic pig density	Head/km^2^	Continuous	0.90 (0.18–7.89)	1.18
Average number of pigs per a smallholder farm	Head	Continuous	1.2 (0–616)	>5
Total volume of live pigs' movements from ASF-affected regions of Russia	Head or yes/no	Continuous or categorical (yes/no)	75 (0–1319)	3.05
Total volume of pig products movements from ASF-affected regions of Russia	kg	Continuous	120 422 (0–7 126 257)	1.35
ASF-affected region bordering with ASF-affected region of Russia	Yes/no	Categorical (yes/no)	0–1	1.54
Proportion of rural population in the total population of the district	%	Continuous	0.8 (0–1)	>5
Forest/total area proportion	%	Continuous	9.8 (0 – 29.7)	>5
Number of ASF cases in wild boar	Number or yes/no	Continuous or categorical (yes/no)	1 (0–7)	>5

## Descriptive Spatial Analysis

To explore the potential relationship between the ASF outbreaks in domestic pigs and wild boar, we used colocation analysis, a geographic information system (GIS) technique (15–17). This technique measures local patterns of spatial association between two categories of point features using colocation quotient statistics. In this study, the local colocation quotient (LCQ) expressed the local proportion of wild boar ASF cases' locations within a defined neighborhood of domestic pig outbreaks' locations. The analyzed locations were then randomly permuted within the entire study area to estimate whether the observed distribution differs from a random distribution and to calculate the *p*-value of the pattern (9,999 permutations were used in our study to achieve the minimum *p*-value of 0.0002). If the local proportion was higher than the global proportion, the LCQ was > 1. As the colocation analysis is not symmetric, the relationships between domestic pig and wild boar outbreaks and wild boar and domestic pig outbreaks were both explored. Neighborhoods for the LCQ calculations were defined as a circle with a radius equal to the mean neighboring distance for the set of ASF outbreaks, calculated using the Average Nearest Neighbor GIS tool. The colocation was analyzed using a time window accounting of 14 days (i) and 45 days (ii) before and after the analyzed outbreaks to add epidemiological meaning to the relationship between ASF outbreaks. Those time periods correspond to an average and maximum duration of infectious period in domestic swine and wild boar as reported elsewhere (18, 19). It was assumed that ASF outbreaks may be epidemiologically related (for example, an outbreak in domestic pigs might be associated with an infected wild boar from a close neighborhood or a wild boar outbreak might be associated with contaminated waste or the improper disposal of domestic pigs' carcasses). Except for the local colocation quotient, a global colocation was also evaluated (GCQ), which expresses a measure of spatial association between both categories of locations across the entire study area.

## Regression Analysis

To identify the ≪susceptibility≫ of districts to ASF outbreaks in domestic pigs, a Generalized Linear Logistic Regression (GLLR) analysis was used (20, 21), where the presence or absence of ASF outbreaks in domestic pigs was considered as a response variable. Several socioeconomic and environmental explanatory variables were used in this analysis, as listed in Table 1. To remove redundant variables, a preliminary analysis for Variance Inflation Factor (VIF) was conducted for the model using a threshold VIF of 5, so that all variables with VIF > 5 were excluded from further modeling.

The model was fitted using stepwise exclusion of the insignificant variables to achieve lowest Akaike information criterion (AIC) value with stepAIC procedure in R programming environment. The significance of variables was evaluated using the Student's *t*-test. The goodness of the logistic regression model fit was evaluated using the proportion of the explained variation in the response variable, and joint Wald statistics, which evaluate the efficacy of independent variables based on a null hypothesis assuming their inefficacy. A Hosmer–Lemeshow test was applied to evaluate an overall goodness of the model's fit by indicating of whether the differences between the expected and observed proportions are significant. The spatial distribution of both response variable and model residuals was evaluated using Moran's I spatial autocorrelation test, which demonstrates compliance of the observed spatial distribution of the analyzed variable to a hypothetical random distribution (null hypothesis). Values of Moran's I coefficient close to zero corresponding to low z-scores with *p* > 0.05 indicate normality of the studied distribution. A presence of spatial autocorrelation in both response variable and residuals would indicate an unexplained clustering of studied phenomenon non adjusted by explanatory variables.

## Space-Time Cluster Analysis

A space-time cluster analysis was conducted using Kulldorff scan statistics (22), which allows for the identification of clusters in the studied area, where disease events (outbreaks) were grouped more densely than could be expected according to the null hypothesis assuming their random distribution. The analysis uses a cylindric moving scan window, where the vertical dimension represents time. A space-time permutations model used in our analysis only evaluates the presence of space-time clustering of the studied features regardless of any background denominator scores (such as population density etc.). The maximum scanning window sizes were chosen as 50% of the size of the study area and 50% of the study period.

## Software

Cleansing, validation, and preliminary evaluation of the data were conducted with Microsoft Office Excel (Microsoft Corporation, Redmond, Washington, U.S.). Preliminary VIF analysis and model fitting were conducted in R programming environment (23) with MASS (24), car (25), and plyr (26) packages. Data were converted into shapefiles for analysis and visualization using GIS-technologies. The spatial and regression analyses were performed using the geographical informational systems ArcGIS Desktop version 10.8.1 and ArcGIS Pro version 2.7 (Esri, Redlands, California, U.S.). SaTScan software (27) was used for the cluster analysis.

## Results

### Descriptive Epidemiological Analysis

The first cases of ASF in the Samara Oblast were reported in the middle of January 2020 in the area bordering the already affected Ulyanovsk Oblast. Despite all disease control measures, the risk of further spreading of the ASF virus in neighboring regions was high. In the Samara Oblast, 41 outbreaks of ASF in domestic pigs and 40 cases in wild boar were reported in 2020 (Figure 1B). Ninety-five percent of the ASF outbreaks in domestic pigs occurred on smallholder farms with the number of susceptible animals ranging from 1 to 140 (median: 16 pigs). The average morbidity rate in these farms was 0.72 ± 0.34, with a mortality rate of 0.57 ± 0.38. The total mortality rate was 0.56 ± 0.38. Outbreaks on two large industrial pig farms with 38,960 and 3,391 susceptible animals resulted in 404 and 538 infected animals, respectively. In wild boar, the incidence ranged from 1 to 30 infected animals, with a median of two.

**Figure 2:**
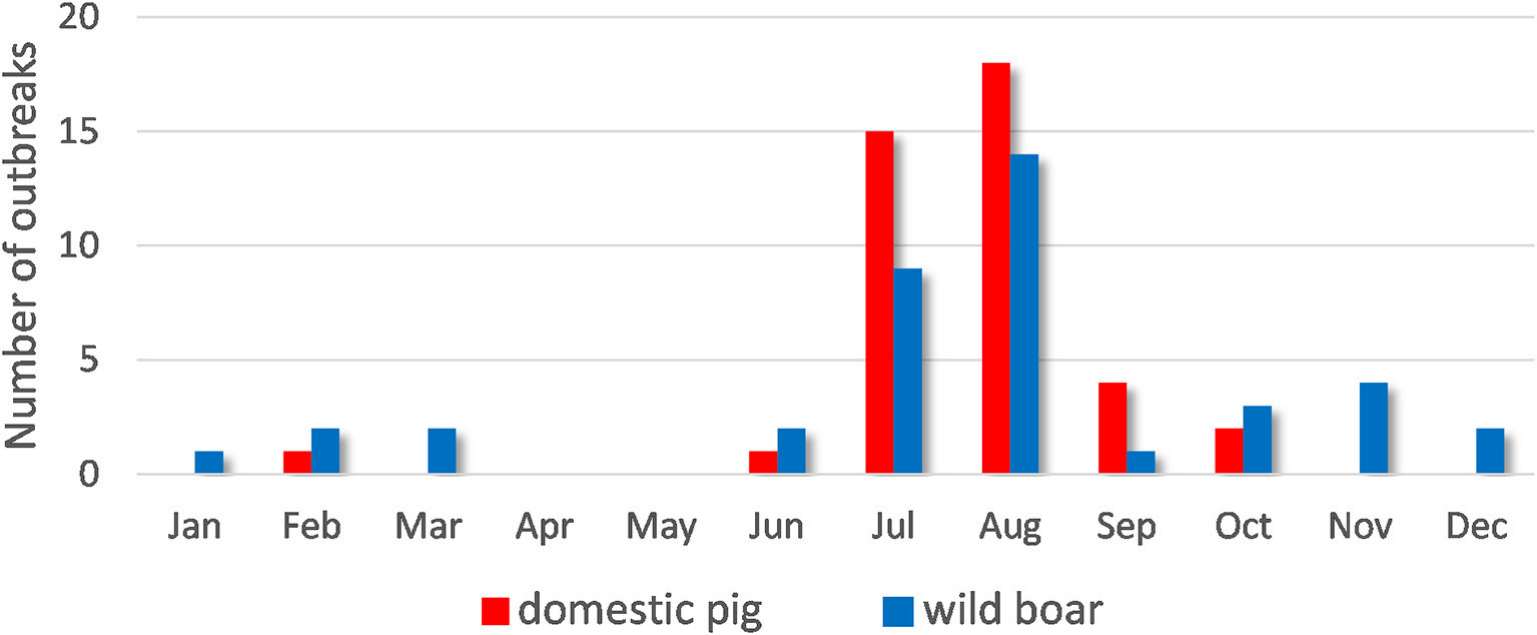
Temporal distribution of African swine fever cases in domestic pigs and wild boar in the Samara Oblast in 2020.

ASF outbreaks were more frequent during the summer months (July and August) (Figure 2). ASF cases in wild boar were reported from January to March and from June to December, with a peak in July and August. ASF infections in domestic pigs were reported from June to October, with a peak in August.

### Spatial and Space-Time Analysis

**Figure 3:**
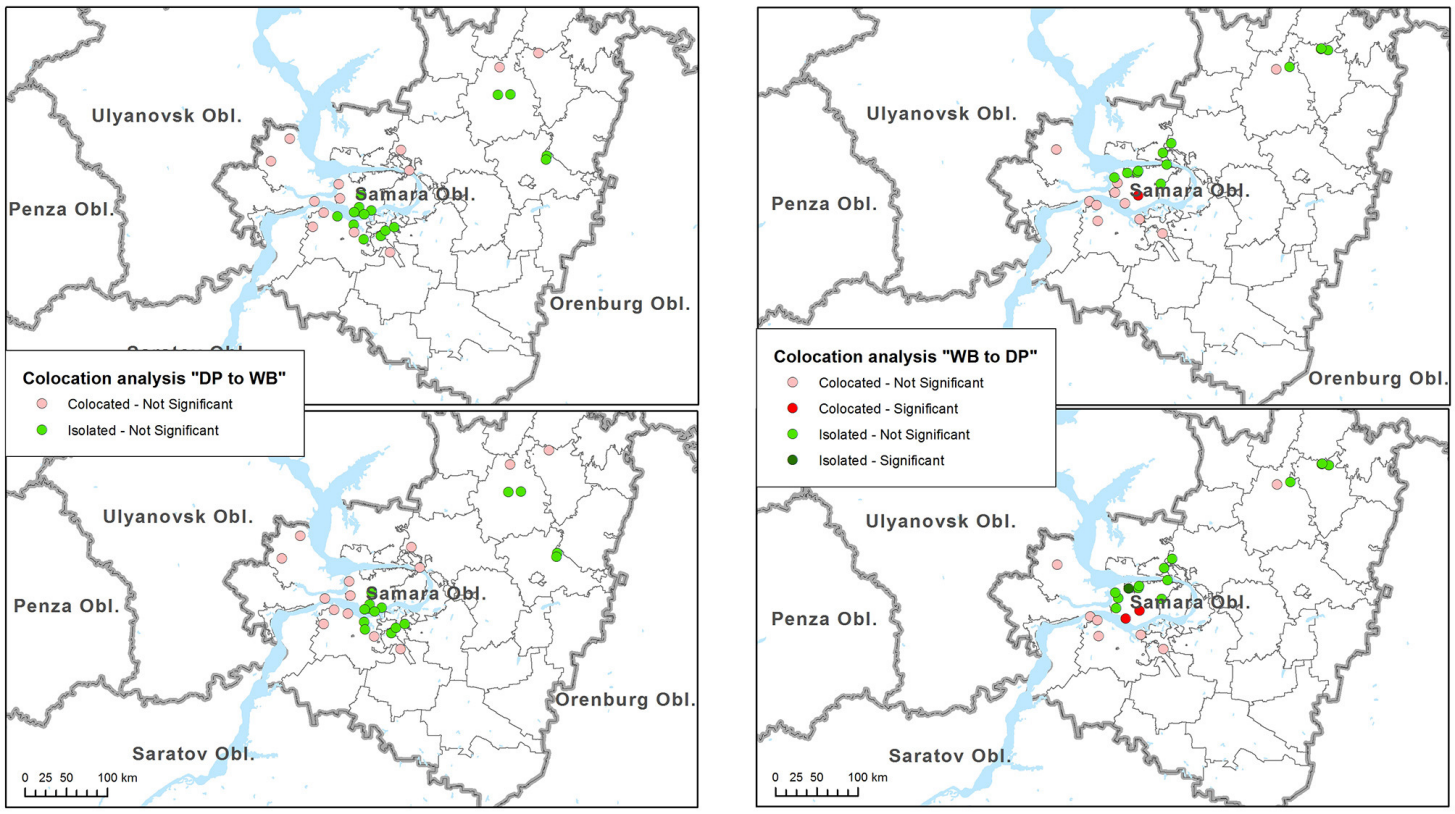
Results of the colocation analysis of African swine fever (ASF) cases in wild boar (WB) near outbreaks in domestic pigs (DP) (left). Colocation of outbreaks in DP near cases in WB (right). Top maps indicate the results for the analysis time span of 14 days, while bottom maps indicated the results for 45-days' time span.

Results of colocation analysis are presented in Table 2. Overall, no evidence of significant global colocation between ASF outbreaks in domestic pigs and wild boar were revealed. At both time windows analyzed, outbreaks in domestic pigs were found to be globally insignificantly collocated with cases in wild boar, while the reverse relationships were found to be insignificantly isolated. No cases in wild boar were found to be significantly concentrated near outbreaks in domestic pigs, neither isolated (Figure 3, left). Similarly, no significant clustering of outbreaks in domestic pigs around cases in wild boar were identified. One and two cases in wild boar (4 and 8%, respectively) were significantly collocated with outbreaks in domestic pigs at 14 and 45 days periods respectively, while one case in wild boar was significantly isolated at 45 days period (Figure 3, right).

**Table 2:** Colocation analysis results^*^.

	**Domestic Pigs to Wild Boar**		**Wild Boar to Domestic Pigs**	
Time span	14 days	45 days	14 days	45 days
Significantly collocated (*p* ≤ 0.05)	–	–	1	2
Insignificantly collocated (*p* > 0.05)	13	14	10	7
Significantly isolated (*p* ≤ 0.05)	–	–	–	1
Insignificantly isolated (*p* > 0.05)	17	16	12	13
Global colocation	Insignificantly collocated (GCQ = 1.05, *p* = 0.63)	Insignificantly collocated (GCQ = 1.14, *p* = 0.22)	Insignificantly isolated (GCQ = 0.93, *p* = 0.64)	Insignificantly isolated (GCQ = 0.94, *p* = 0.61)

* *those locations having no neighbors within the defined neighborhood radius were removed from the analysis*.

The preliminary data analysis revealed significant correlations between the population density and road density (*r*_s_ = 0.88), and the human population density and proportion of rural population (*r*_s_ = −0.74). Therefore, population density was excluded from the regression analysis. Further calculation of VIF for the model left the following predictors with VIF ≤ 5 (Table 1):

a. wild boar density,
b. summary pig population,
c. pig population density,
d. smallholder farms density,
e. summary road length,
f. volume of pork products' importation from ASF-affected regions of Russia,
g. importation of live pigs from ASF-affected regions of Russia,
h. presence of a common border with an ASF-affected region.

**Figure 4:**
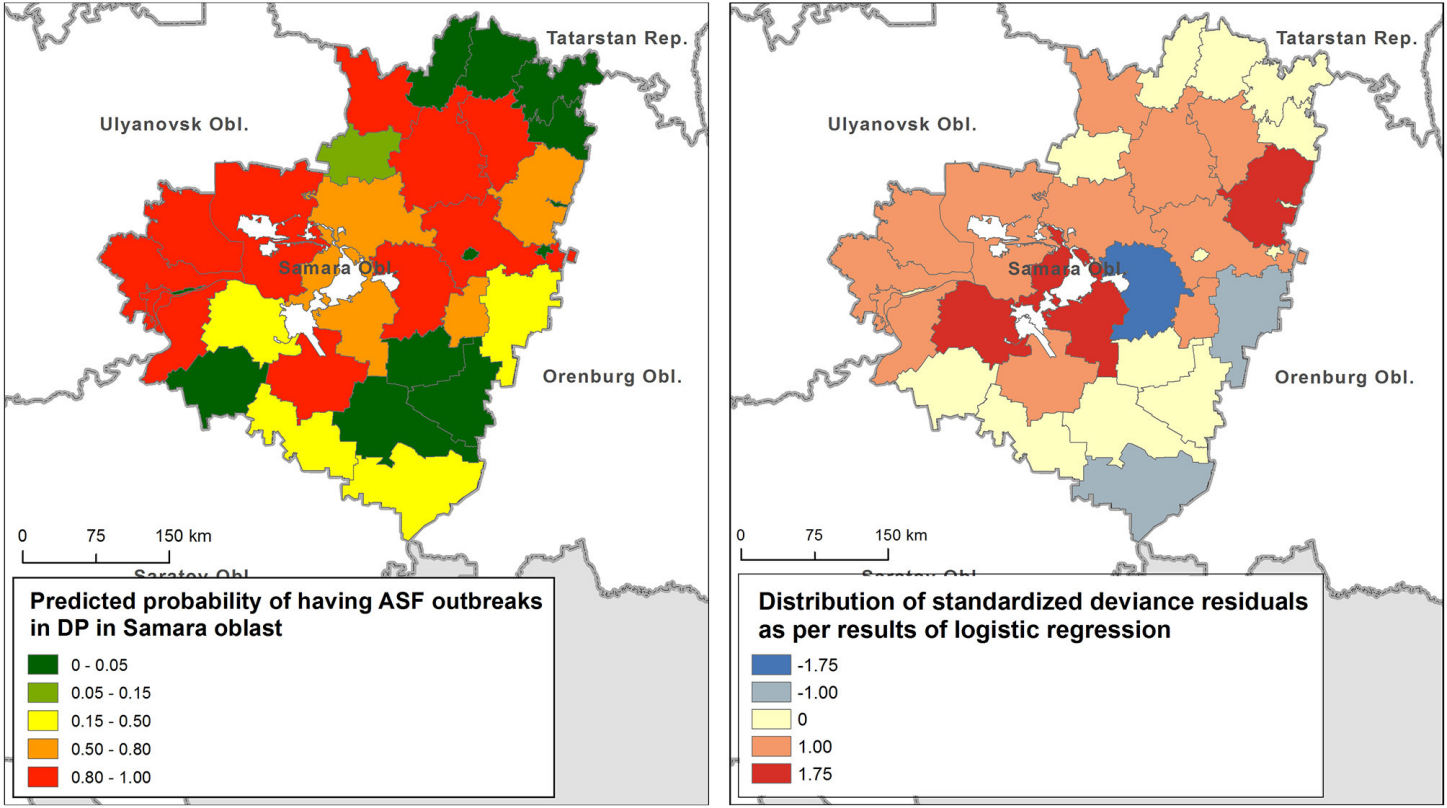
Predicted probability of having an African swine fever (ASF) outbreak in domestic pigs (DP) in the Samara oblast (left), and the distribution of deviance residuals (right).

The final GLLR model includes: importation of live pigs from ASF-affected regions of Russia as the most significant variable (*p* < 0.05), volume of pork products' importation from ASF-affected regions of Russia, density of smallholder farms and total pig population of the district as less significant factors (*p* < 0.1), while presence of common border with ASF-affected region was found to be insignificantly associated with ASF infection status of the district (*p* > 0.1). This model had the lowest AIC, statistically significant Wald test (*p* < 0.0001), and acceptable share of explained variation of 0.67. A Hosmer-Lemeshow test returned χ^2^ = 6.11 with *p* = 0.63, thus suggesting an overall model significance. The distribution of the predicted probability of having ASF outbreaks in domestic pigs is shown in Figure 4 (left). Table 3 presents the regression metrics for the model. The test for spatial autocorrelation of the response variable returned a Moran's I index of −0.02 that corresponds to z-score of 0.06, *p* =0.94. For the model residuals, Moran's I index was estimated as −0.13, z-score = −0.73, *p* = 0.46. Hence, no spatial autocorrelation was found in response variable, neither in residuals (Figure 4, right). This suggest no spatial dependencies existed in the data distribution that would not be unexplained by the model.

**Table 3:** Logistic regression metrics.

**Independent variable**	**Logistic regression coefficient**	**Standard error**	***p*-value**	**OR**	**OR 95% CI**
Intercept	−8,71	4,48	0,05	0,0001	0.0000–1.0832
Live pig movements from ASF affected RF regions	5.917	2.785	0.03	371.52	1.58–87290.57
Volume of pork products' movements from ASF-affected regions	4E-6	2E-6	0.06	1.001	1.000–1.002
Summary pig population	0.002	0.001	0.09	1.002	0.999–1.004
Density of smallholder farms	1.078	0.653	0.09	2.941	0.817–10.585
Shared border with ASF affected region	4.49	3.64	0.21	89.18	0.07–11,208.64

*OR, odds ratio; CI, Confidence Interval*.

The only statistically significant (*p* < 0.001) cluster of ASF outbreaks was identified in the southern part of the region (including the Neftegorsky, Krasnoarmeysky, Bolsheglushitsky, and Volzhsky districts) (Figure 1B). Four outbreaks of ASF were reported from September 18 to October 8 in this area with a radius of 40 km. The first outbreak involved domestic pigs while the rest of cases were in wild boar.

## Discussion

This study presents an epidemiological analysis of ASF outbreaks in domestic pigs and wild boar in the Samara Oblast using spatial and colocation analyses. A regression model was used to evaluate the influence of socioeconomic factors on the occurrence of ASF in domestic pigs. Despite the fact that the supposed dependent variable (number of ASF outbreaks notified in domestic pigs) represents count data, its distribution was zero-inflated with comparatively low variation (var = 4), and the number of outbreaks may have been underreported due to attempts by householders to disguise ASF cases in their farms (28–31). Therefore, a logistic model that interprets the dependent variable as the presence or absence of outbreaks was used.

No evidence of significant spatio-temporal associations between outbreaks in domestic pigs and those in wild boar were identified in this study. These results do not allow us to accept a hypothesis of direct epidemiological correlation between nearby outbreaks in wild boar and domestic pigs, and suggest that in this epidemic a close contact between susceptible wild and domestic pigs could hardly play a predominant role in ASF transmission. A similar conclusion is supported by the fact that there was no tendency revealed for ASF outbreaks to cluster in a major part of the region, though the results of colocation and spatio-temporal cluster analyses may be influenced by the underestimation of the number of cases in wild boar due to underreporting. However, an overlap between the dynamics of ASF outbreaks in wild and domestic suids was observed (Figure 2). This overlap demonstrates the indirect influence of the populations on one another. ASF cases in wild boar can be related to the end of hunting seasons and breeding periods. The prevalence of outbreaks in domestic populations is likely related to human activities (agricultural activities, trade, and economic relations) and human visits to wild boar habitats and feeding grounds (such as mushroom gathering or berry picking). Though, the results of spatio-temporal analysis should be interpreted carefully because they are subject of reporting accuracy, while underreporting of cases in wild boar may be assumed. Thus, it is believed that as maximum as 10 percent of wild boar carcasses are normally found (32).

The main factors related to the occurrence of ASF outbreaks in domestic pigs identified in this study include the transportation of pigs and pork products from previously infected regions, summary pig population, density of smallholder farms, and sharing borders with ASF-affected regions. These results are consistent with previous studies of risk factors for livestock infections in smallholder farms and studies regarding factors contributing to intraregional infection transmission. The introduction of ASFV via the shipment of pigs was found to be the most significant risk factor for ASF transmission in other studies (33, 34). Sharing a border with a previously-infected region increases the risk of infection during the local movement of people and domestic pigs and the migration of wild boar (35), though it was found to be statistically insignificant in our study. The summary pig population and density of smallholder farms were also identified as significant factors associated with the ASF presence in a district providing an indication of a local pig farming system density that promotes between-holdings contacts and facilitates the ASF transmission (36, 37).

The results of this study indicate that human-mediated activities, and the intensity of smallholder pig operations may be the main driving force of the ASF epidemic in the Samara Oblast independent of the density of wild boar. More studies are required to identify additional risk factors and to clarify a mutual influence of wild boar and domestic pigs populations in order to develop a risk map as a basis of a prognostic model of ASF spread in regions of the Russian Federation and other countries with high proportion of rural inhabitants that are currently free from ASF. A colocation analysis presents an interesting GIS technique that enables studying the space-time relationships between ASF cases in domestic and wild pigs, and provides further opportunities for deeper understanding of observed epidemiological patterns of the disease local spread.

This study is limited by the incomplete assessment of factors associated with a lack of statistical data. For example, the contribution of the illegal sale and movement of pigs and pork products to the spread of ASF cannot be assessed. Data regarding movements between districts within the Samara Oblast are also missing.

In conclusion, this study identifies the spatio-temporal patterns and epidemiological associations of ASF outbreaks in the Samara Region of the Russian Federation in 2020. No obvious associations between outbreaks in domestic pigs and wild boar were identified. ASF-infected districts were associated with the transportation of live pigs from ASF-affected regions of Russia, suggesting socioeconomic links as the main factor of disease spread within the region. The results clearly underline the importance of considering animal transportation data as an explanatory factor in further modeling efforts.

## Data Availability Statement

The original contributions presented in the study are included in the article/Supplementary Material, further inquiries can be directed to the corresponding author.

## Author Contributions

AGl, DL, and AGo: conceptualization. FK, OZ, AB, and AGo: methodology. AGl, FK, DL, and OZ: formal analysis. TS, AB, and AGo: validation. AGl, FK, DL, and AK: data curation. FK, AGl, DL, and TS: writing-original draft preparation. FK, TS, OZ, AB, and AGo: writing-review and editing. AGl and DL: visualization. AB and AGo: project administration. All authors have read and agree to the published version of the manuscript.

## Conflict of Interest

The authors declare that the research was conducted in the absence of any commercial or financial relationships that could be construed as a potential conflict of interest.

## Publisher's Note

All claims expressed in this article are solely those of the authors and do not necessarily represent those of their affiliated organizations, or those of the publisher, the editors and the reviewers. Any product that may be evaluated in this article, or claim that may be made by its manufacturer, is not guaranteed or endorsed by the publisher.
