# Supplementary material for: Risk Factors of African Swine Fever in Domestic Pigs of the Samara Region, Russian Federation
Source: Front Vet Sci. 2021 Aug 24;8:723375. doi: 10.3389/fvets.2021.723375 (PMC8421595; doi:10.3389/fvets.2021.723375)
Supplement: Supplementary file 1 [file Data_Sheet_1.pdf]

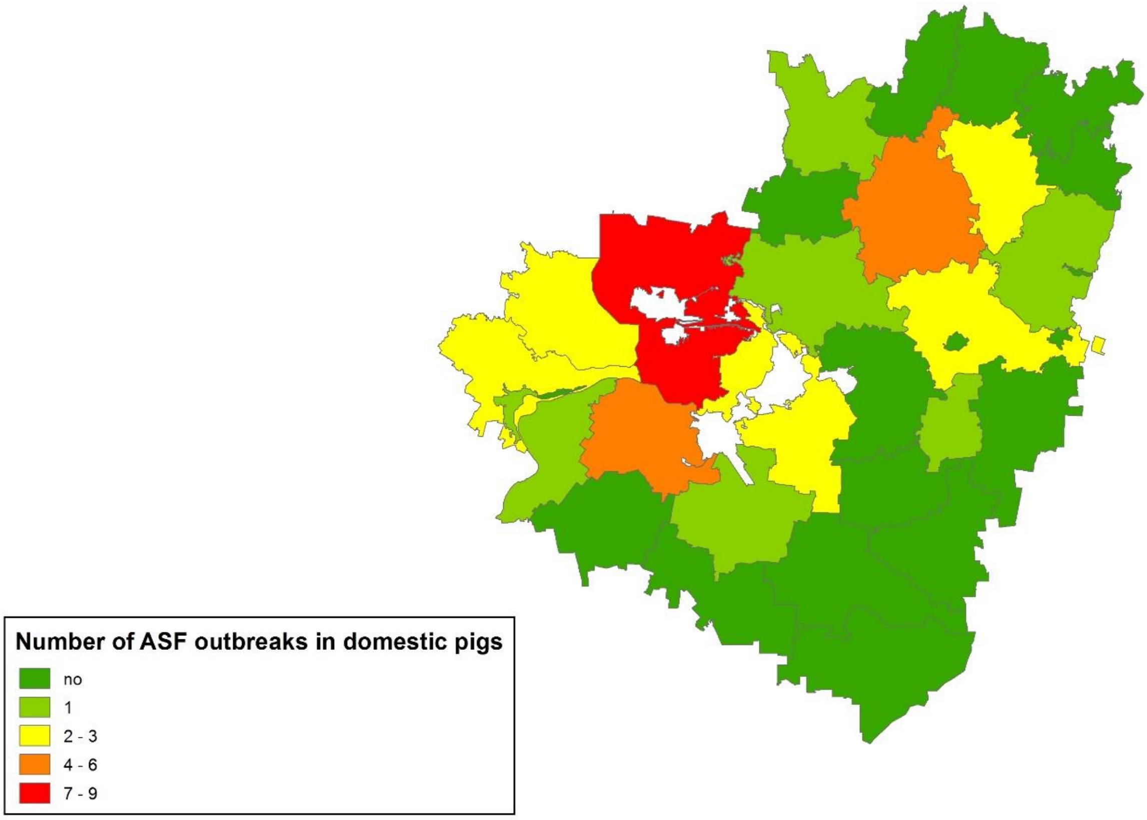

Supplementary Figure 1. Number of ASF outbreaks in domestic pigs.

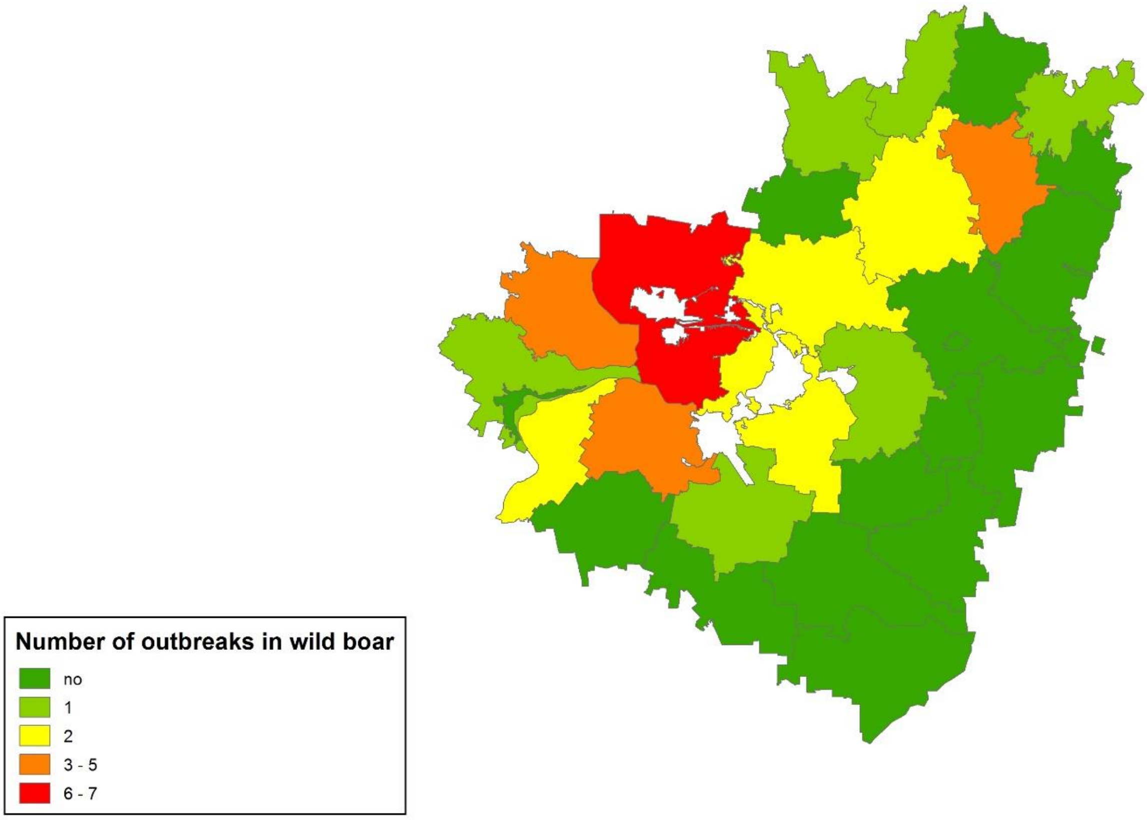

Supplementary Figure 2. Number of ASF outbreaks in wild boar.

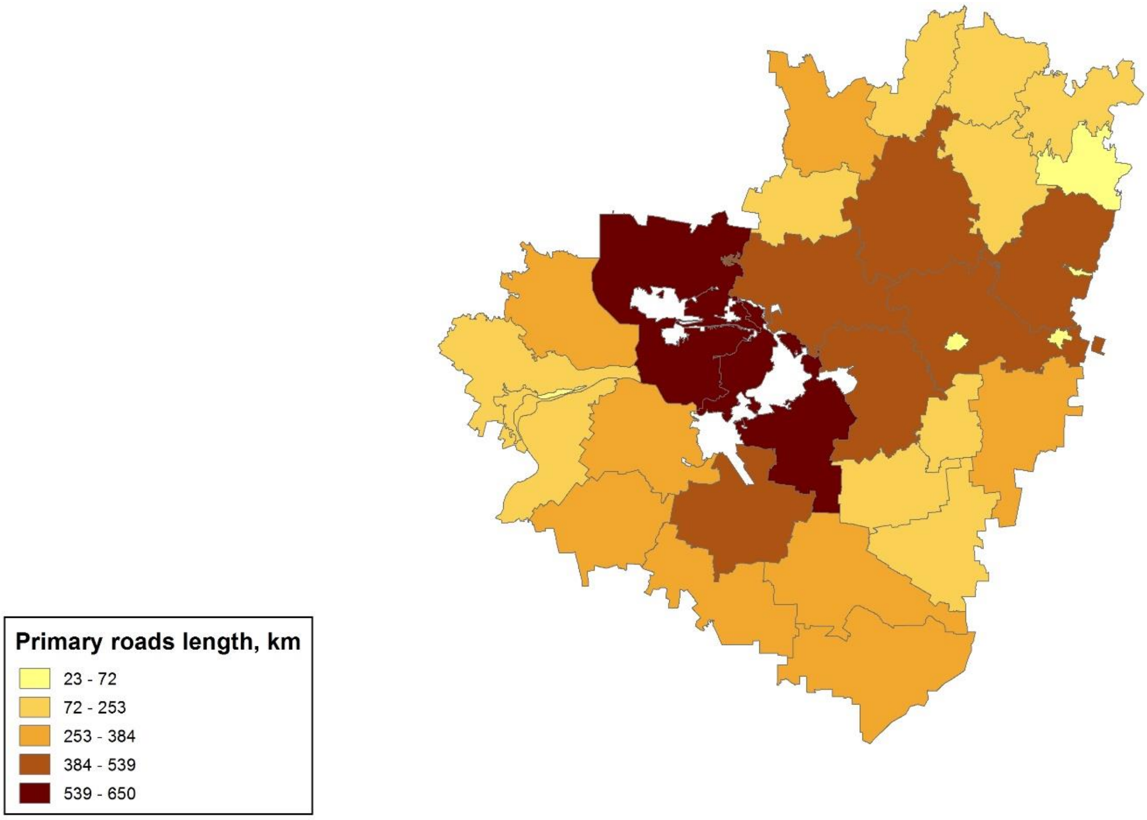

Supplementary Figure 3. Primary roads length, km.

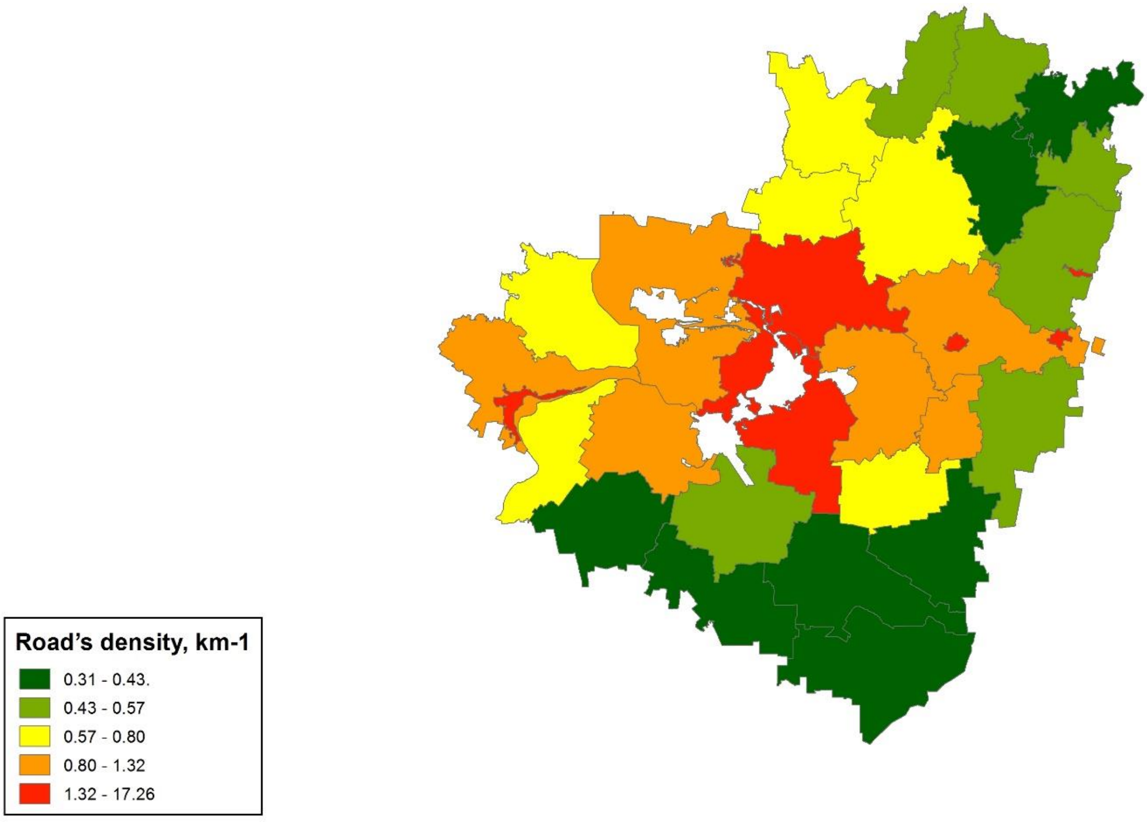

Supplementary Figure 4. Road's density, km^{-1}.

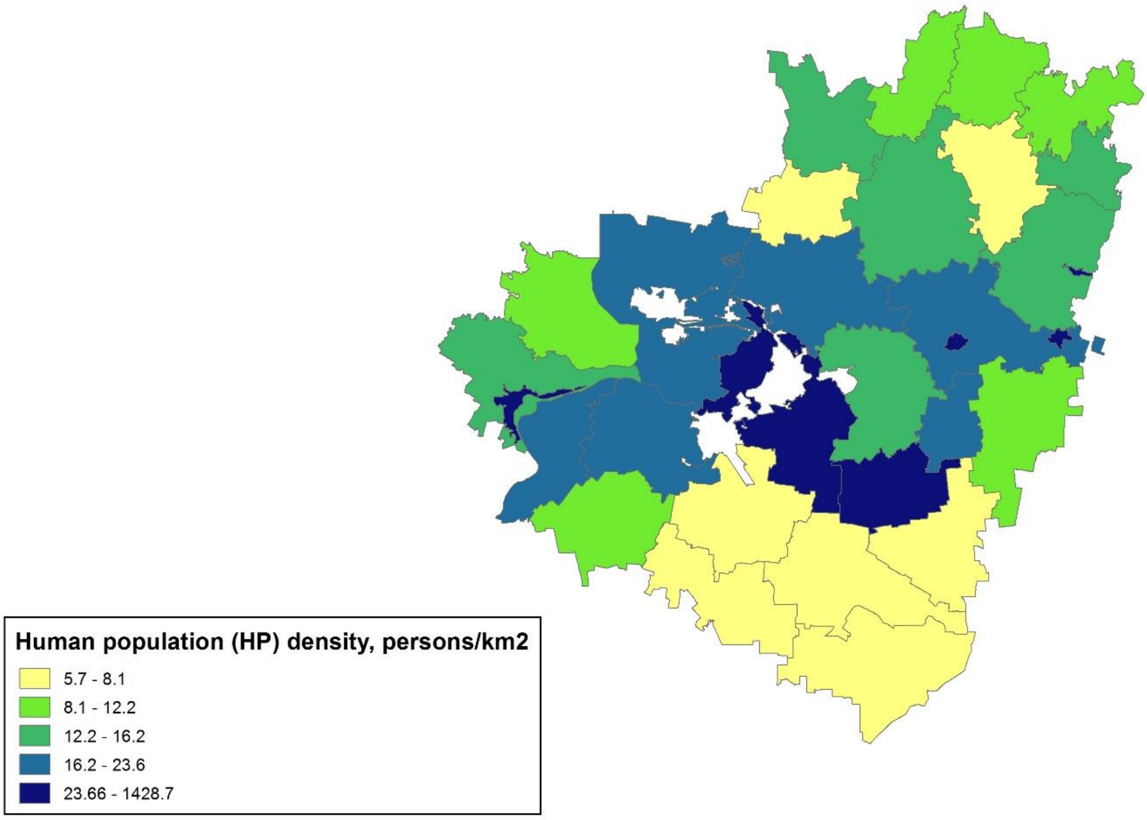

Supplementary Figure 5. Human population (HP) density, persons/km^2.

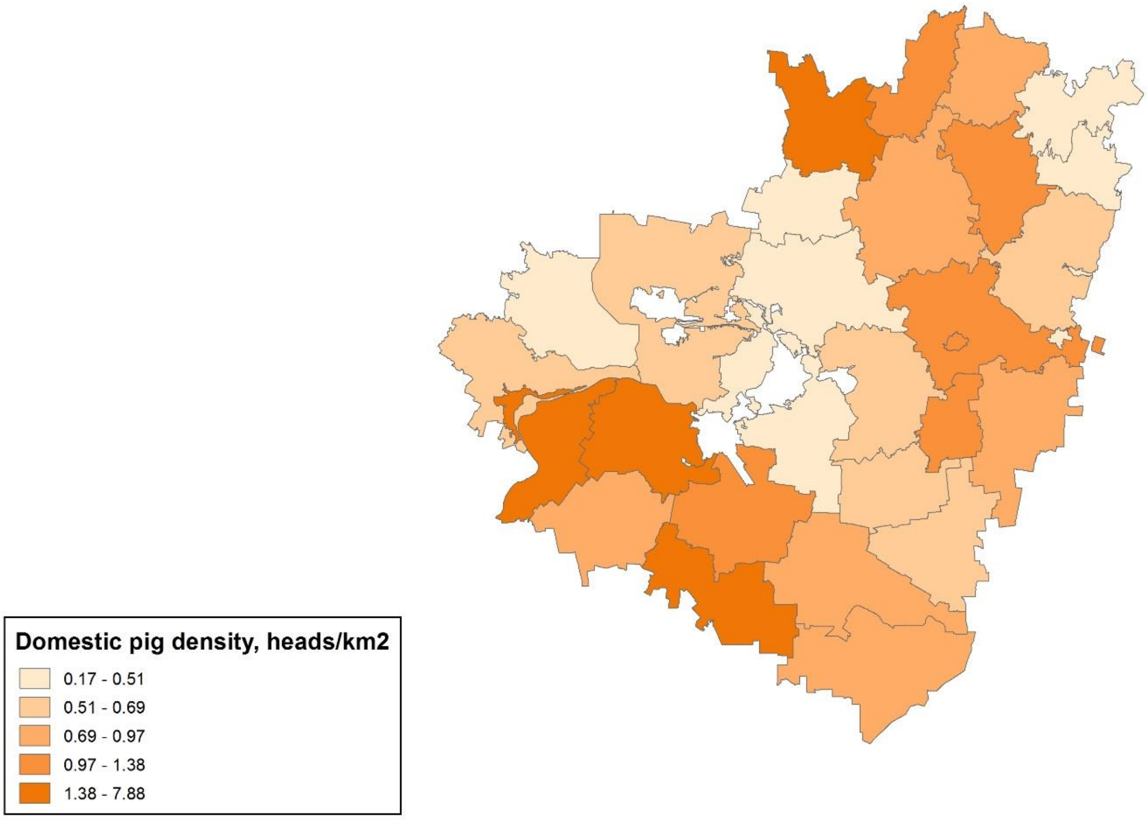

Supplementary Figure 6. Domestic pig density, heads/km^2.

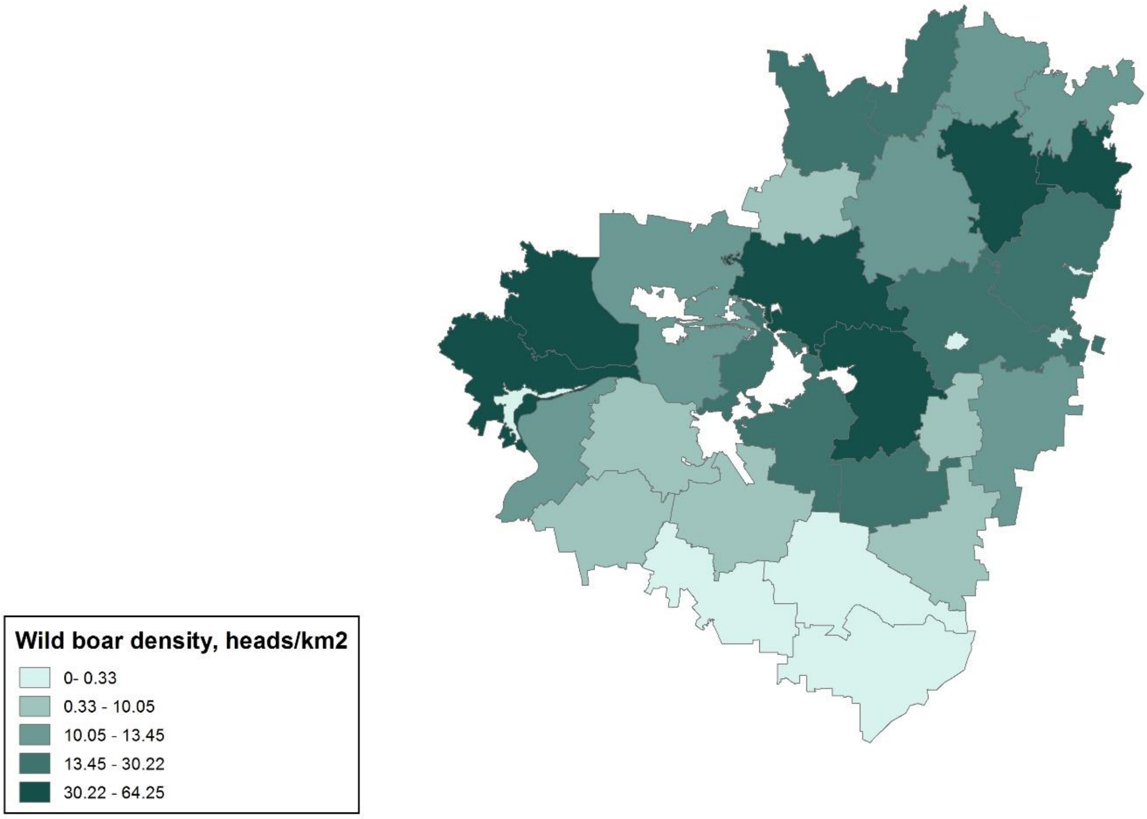

Supplementary Figure 7. Wild boar density, heads/km^2.

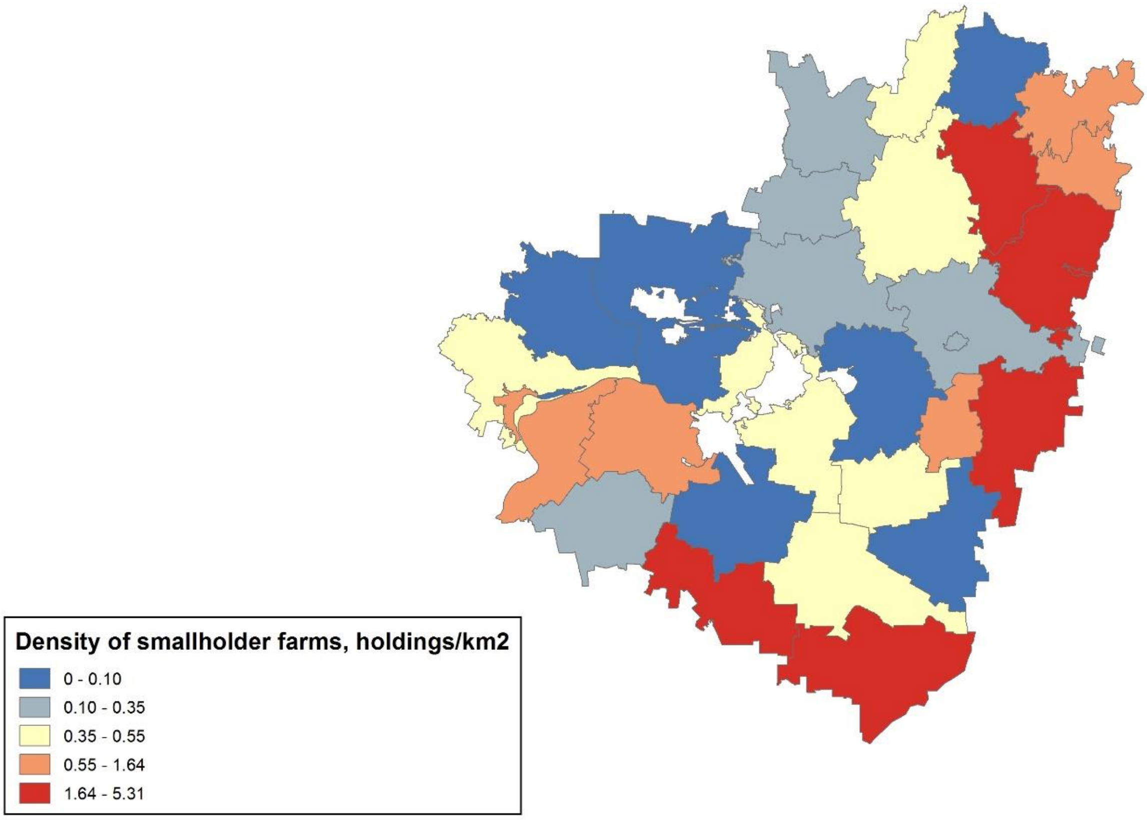

Supplementary Figure 8. Density of smallholder farms, holdings/km^2.

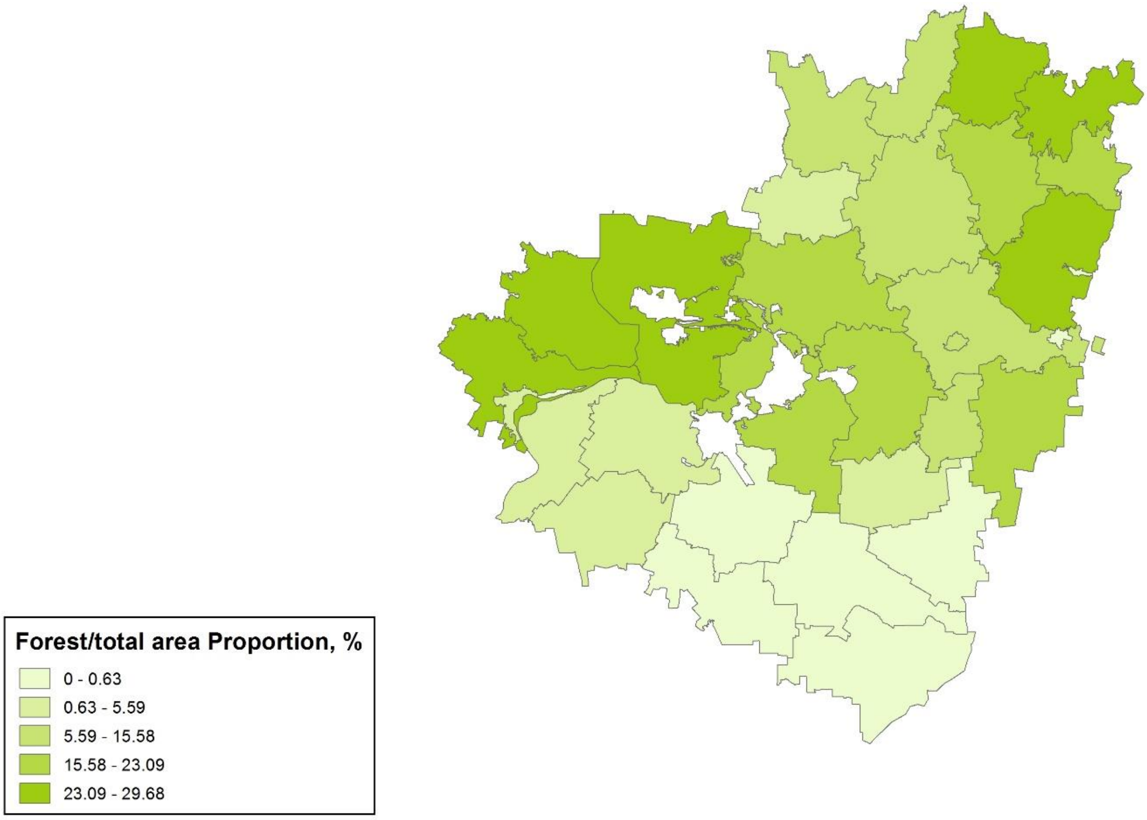

Supplementary Figure Fig. S9. Forest/total area Proportion, %.

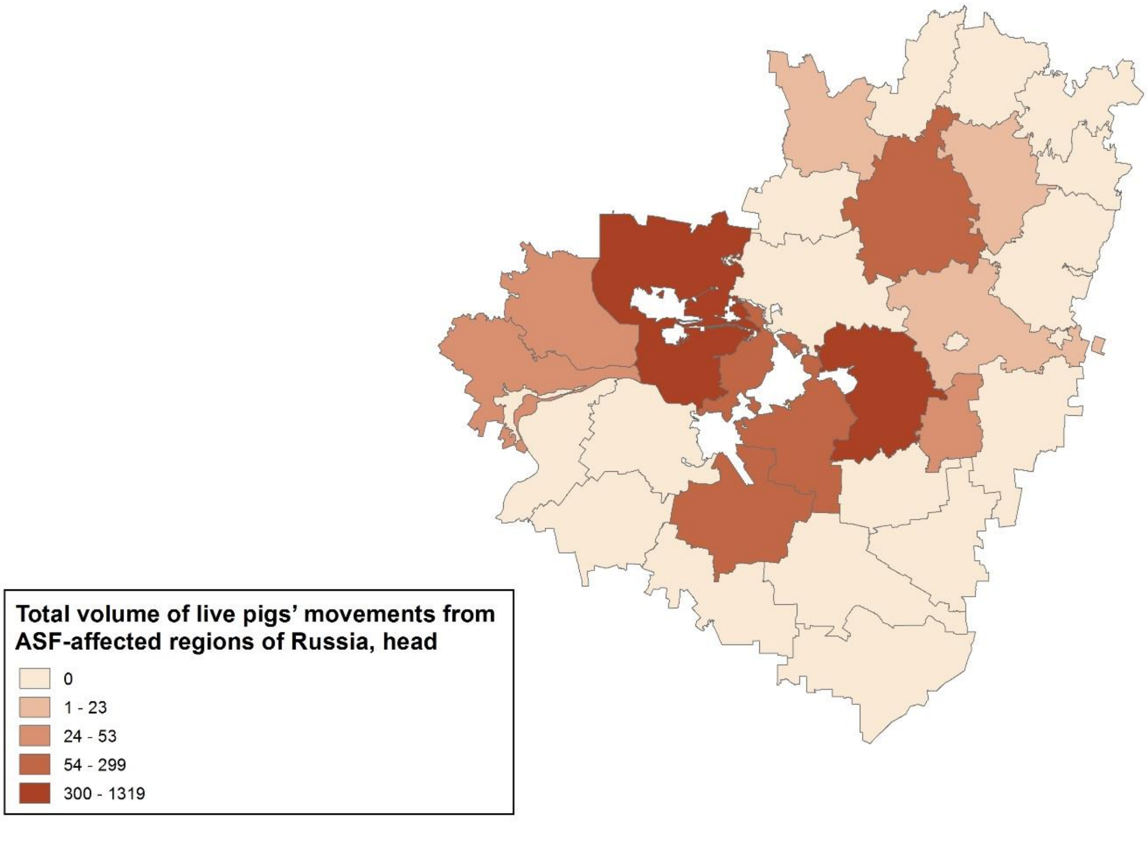

Supplementary Figure 10. Total volume of live pigs' movements from ASF-affected regions of Russia, head.

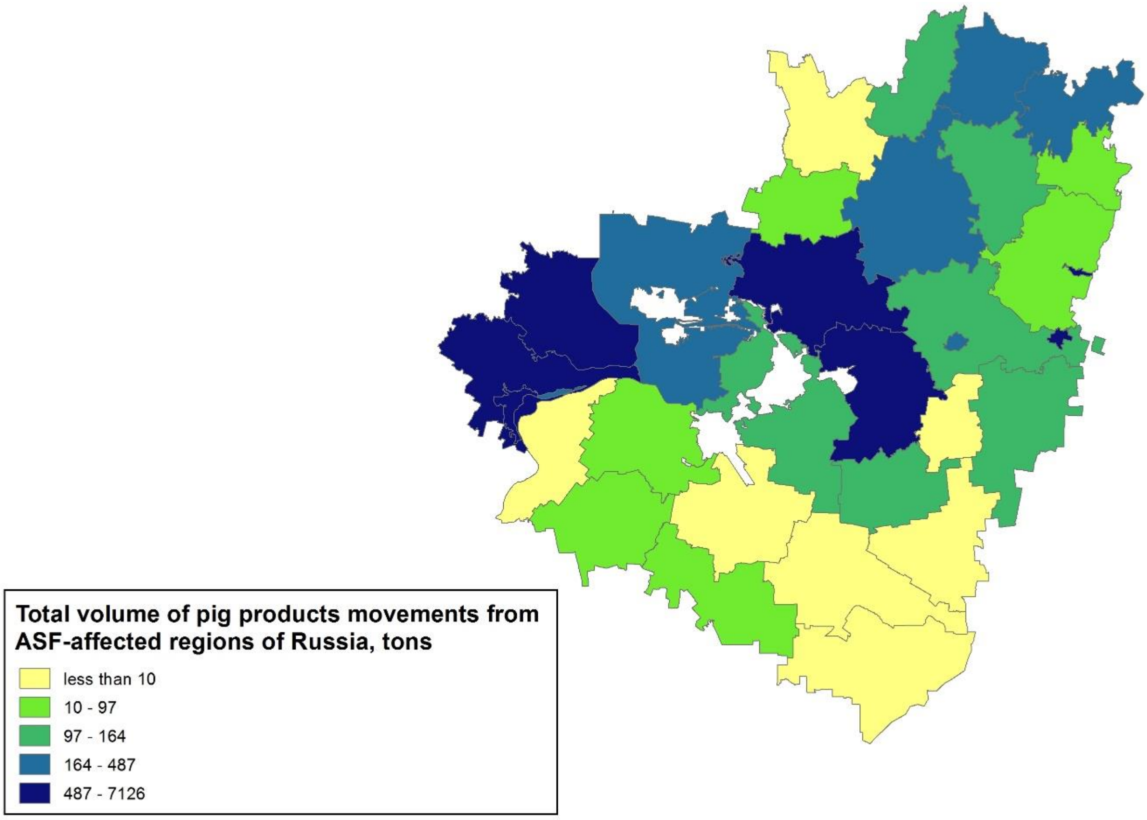

Supplementary Figure 11. Total volume of pig products movements from ASF-affected regions of Russia, tons.

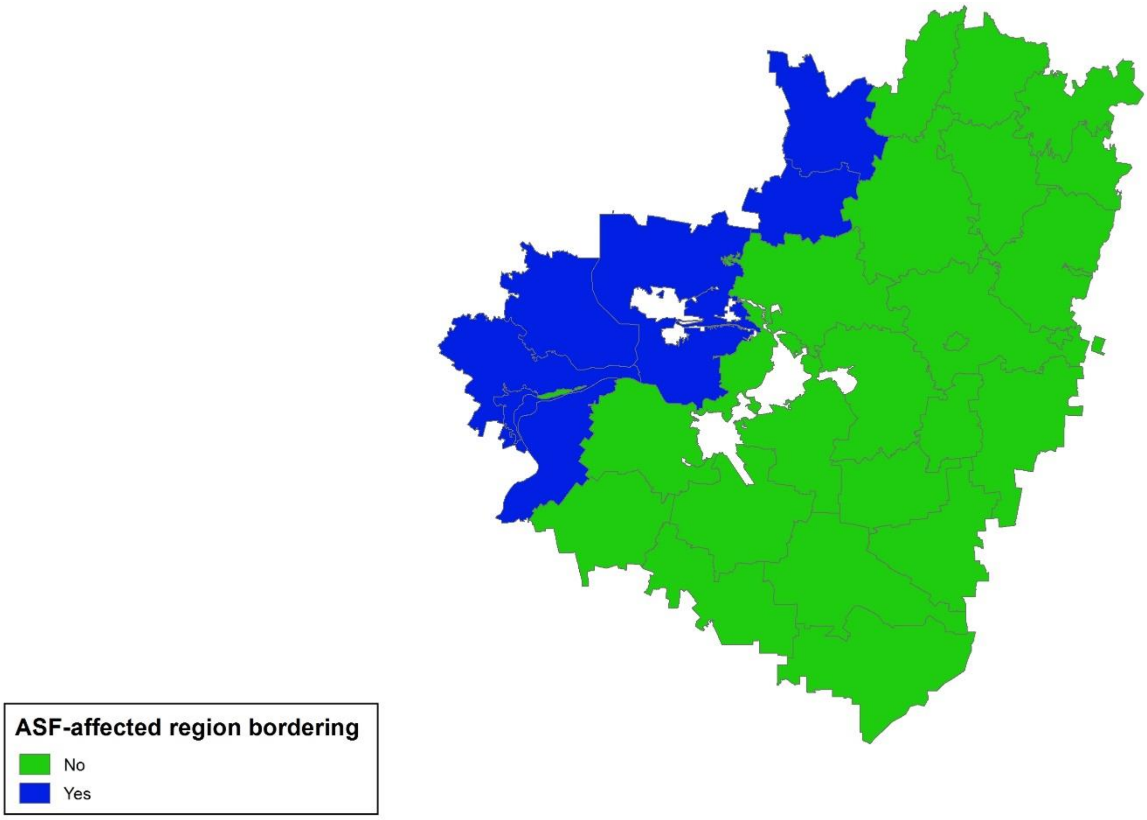

Supplementary Figure 12. ASF-affected region bordering.

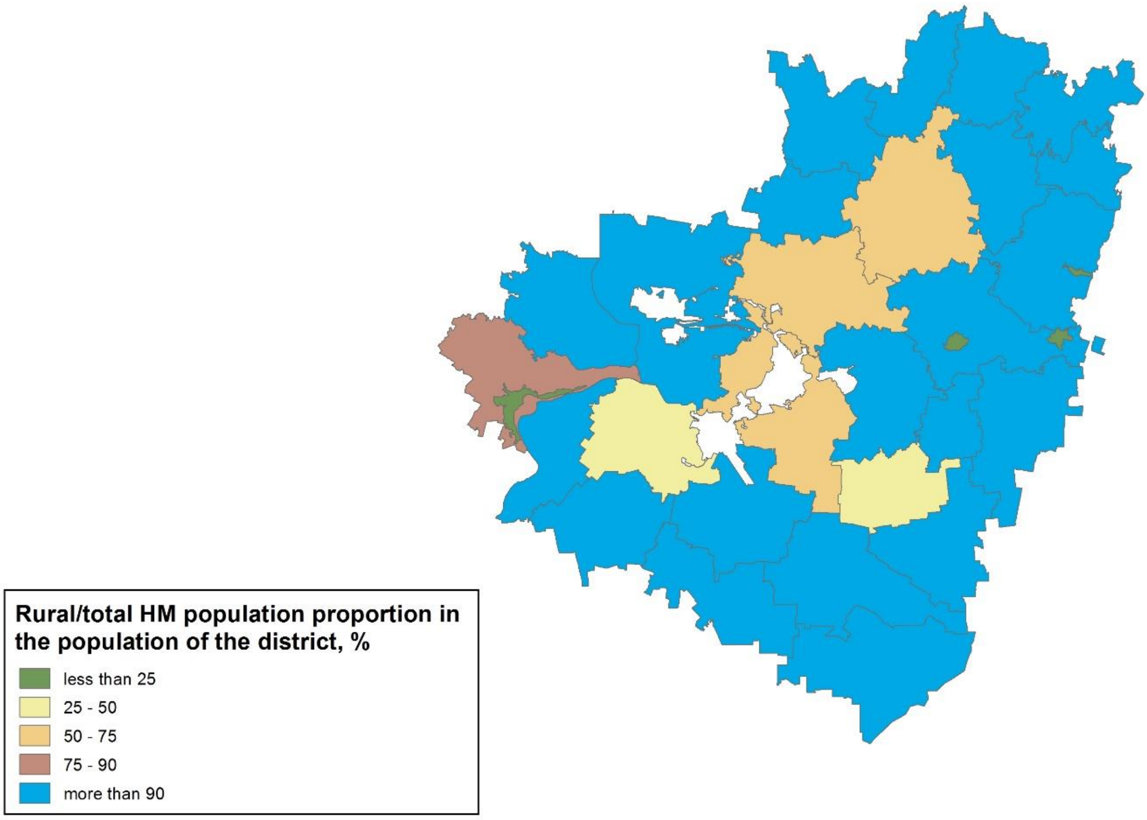

Supplementary Figure 13. Rural/total HM population proportion in the population of the district, %.

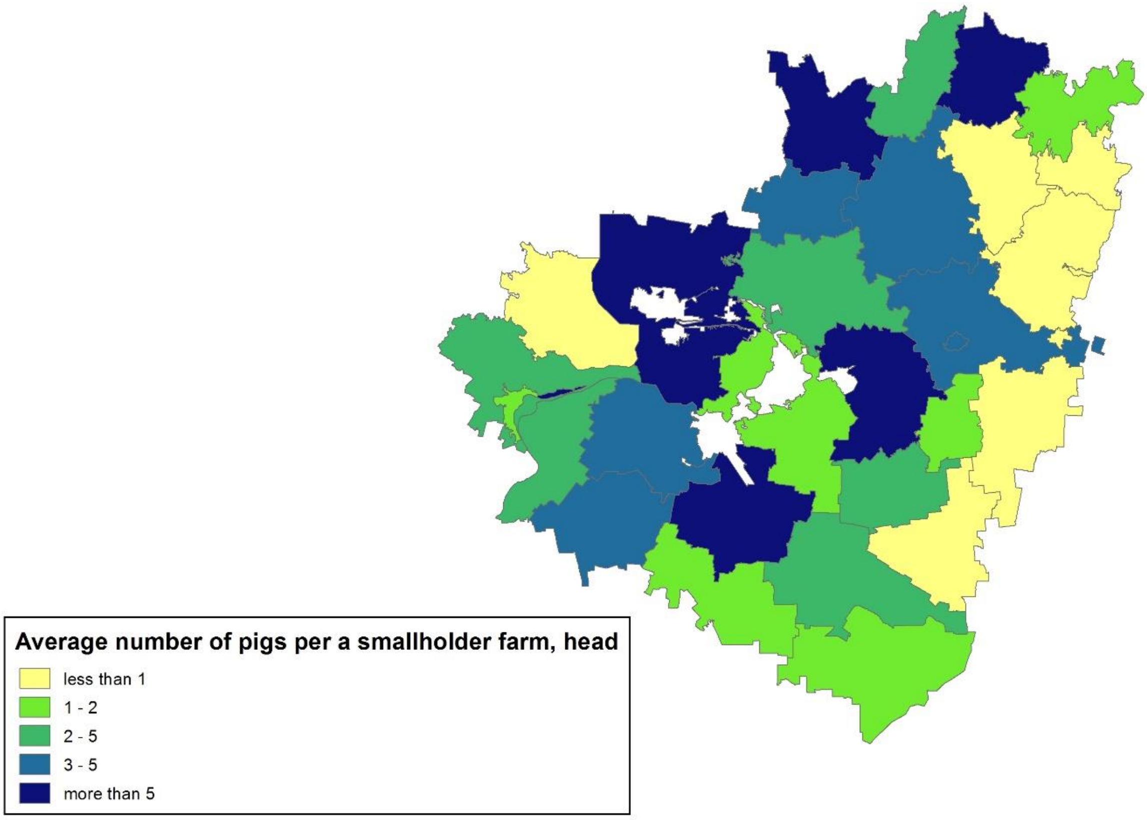

Supplementary Figure 14. Average number of pigs per a smallholder farm, head.
